# With low ovarian reserve, Highly Individualized Egg Retrieval (HIER) improves IVF results by avoiding premature luteinization

**DOI:** 10.1186/s13048-018-0398-8

**Published:** 2018-03-16

**Authors:** Yan-Guang Wu, David H. Barad, Vitaly A. Kushnir, Qi Wang, Lin Zhang, Sarah K. Darmon, David F. Albertini, Norbert Gleicher

**Affiliations:** 1 The Center for Human Reproduction, New York, NY 10021 USA; 2 The Foundation for Reproductive Medicine, New York, NY 10021 USA; 3 Department of Obstetrics and Gynecology, Wake Forest University, Winston Salem, NC 27106 USA; 4 Stem Cell Biology and Molecular Embryology Laboratory, The Rockefeller University, New York, NY 10065 USA; 5 Department of Obstetics and Gynecology, University of Vienna School of Medicine, 1090 Vienna, Austria

**Keywords:** In vitro fertilization (IVF), Advanced female age, Premature ovarian aging (POA), Occult primary ovarian insufficiency (oPOI), Pregnancy rates, Premature luteinization

## Abstract

**Background:**

Highly Individualized Egg Retrieval (HIER), defined as age-specific early oocyte retrieval (ER), has been demonstrated to avoid premature luteinization in women ≥43. We here investigated whether HIER also applies to younger women with premature ovarian aging (POA), and what best lead follicle size should be for administration of ovulation-triggers.

**Methods:**

Fifty-six women ≥43, and 37 POA patients underwent IVF cycles. Granulosa cells (GCs) were isolated, cultures were established, RNA was extracted and real-time PCR analyses performed, with gene expressions at mRNA level investigated for FSH receptor (*FSHR*), luteinizing hormone receptor (*LHCPR*), P450 aromatase (*CYP19a1*) and progesterone receptor (*PGR*). POA was defined by age < 40, FSH above 95%CI and/or AMH below 95%CI for age. Women ≥43 years were divided into very early retrieval (VER), with human chorionic gonadotropin (hCG) trigger at 13.5–15.5 mm, ER at 16.0–18.0 mm or standard retrievel (SR) at 18.5–20.5 mm; POA patients were divided into ER and SR. Pregnancy rates and and molecular markers of premature luteinization (PL) were main outcome measures.

**Results:**

ER resulted in a significantly higher clinical pregnancy rate (16.7%) than VER (5.9%) or SR (6.7%; both *P* < 0.05). Molecular markers of PL were highest with SR and lowest with VER. In POA, ER improved pregnancy chances even more than in women ≥43 (7.7% with SR vs. 41.7% with ER), while also reducing molecular markers of PL. With low ovarian reserve (LOR), by avoiding PL, ER with hCG trigger at 16.0–18.0 mm, thus, improves clinical pregnancy rates at all ages. As VER demonstrated lowest molecular PL marker but equally poor pregnancy rates as SR, too early ovulation triggers, likely, result in cytoplasmatic immaturity.

**Conclusions:**

HIER is even more effective in POA patients than women above age 43, demonstrating that HIER should be further investigated going into even more advanced ages.

## Background

Pregnancy and live birth rates in association with in vitro fertilization (IVF) decline slowly and persistantly with advancing female age [1]. After age 43, this gradual decline, however, significantly accellerates [2]. Based on national outcome reporting, our center serves a disproportionally older patient population in comparison to all other U.S. IVF centers [1]. Quickly declining pregnancy rates after age 43, therefore, have been a long-term research interest at our center, and have been an important subjec of many investigations.

Through molecular in vivo and in vitro studies of granulosa cells (GCs) in patients above age 43, we discovered that follicles at those ages to significant degrees prematurely luteinize (PL) [3]. Ovulation induction with human chorionic gonadotropin (hCG) triggers at standard lead follicle sizes of 19.0–22.0 mm, therefore, produced overmature (i.e., atretic) and otherwise poor quality oocytes and negatively affected clinical IVF outcomes. Earlier hCG triggers at approximately 16.0 mm, and earlier oocyte retrievals, however, avoided effects of PL, thereby improving oocyte quality and clinical IVF pregnancy rates [3]. To describe the age-specific timing of ovulation triggering in IVF, we coined the acronym Highly Individulaized Egg Retrieval (HIER).

Trigger at lead follicle size 16.0 mm in our initial study of HIER was chosen arbitrarily. This raised the follow up question, whether 16.0 mm lead follicle size is really uniformly the best option at all ages to avoid PL. The first purpose of here presented study was, therefore, to find an answer to this question.

A second goal of this study was, however, the determination whether women with premature ovarian aging (POA), by some also called occult primary ovarian insufficiency (oPOI) [4] also prematurely luteinize their follicular environment. Approximately 10% of women in reproductive years suffer from POA/oPOI (5). They, however, are disproportionally concentrated in fertility center. Like older women, they are characterized by abnormally (low age-specific) functional ovarian reserve (LFOR) parameters, including abnormally high follicle stimulating hormone (FSH) and/or abnormally low anti-Müllerian hormone (AMH) [5]. In preparation for this study, we, therefore, hypothesized that, like older women above age 43, by receiving early hCG triggers and early retrievals (ER), patients with POA/oPOI may also benefit from HIER. Here presented evidence confirms this hypothesis.

## Methods

### Patients

This study involved two patient populations: To determine whether hCG triggers had to be administered at progressively smaller lead follicle sizes, we stratified 56 older women above age 43 (range 43–49) to “best” lead follicle sizes at time of ovulation trigger: Very early retrieval (VER) was defined as 13.5–15.5 mm, *n* = 17; ER, as 16.0–18.0 mm, *n* = 24; and standard retrieval (SR) as 18.5 mm- ≥20.5 mm, *n* = 15.

In 37 younger women with POA/oPOI undergoing IVF, we here investigated whether, like older women over age 43 [3], they also demonstrated evidence of PL in their follicles. As previously reported [2], POA/oPOI was defined by LFOR, based on abnormally elevated age-specific FSH and/or abnormally low AMH levels outside of normal age-specific 95% CIs.

### IVF cycle management

IVF cycle managements of women with LFOR are uniform at our center, whether LFOR is due to advanced female age or POA/oPOI: As previously reported [2, 3], all patients received the same maximal dosage of gonadotropin stimulation with FSH (300-450 IU) and human menopausal gonadotropin (150 I.U) daily after ovaries for 6–8 weeks had been prepared for stimulation by supplementation with dehydroepiandrosterone (DHEA) [6] and CoQ10 [7]. Ovulation was triggered with 10,000 IU of hCG I.M.

Older women receiving SR were random long distance patients, monitored locally without access to early triggers. Those patients from cycle day-2 on received a microdose of GnRH-agonists. Though effects of such microdose agonist treatments on here reported results cannot be ruled out completely, they apear very unlikely since microdose agonists are administered to prevent premature luteinization and premature ovulation. Should this medication have affected here reported study outcomes, the microdose agonist protocol, therefore, indeed should have reduced molecular evidence of premature luteinization and, thereby, have biased study outcomes against here reported outcome differences between groups.

Since ER avoids premature ovulation risks, patients scheduled for ERs and VERs did not require either agonist or antagonist treatments to prevent spontaneous ovulation. Table 1 demonstrates that patient characteristics, age, FSH and AMH, did not differ between the three studied patient groups.

**Table 1:** Patient and IVF cycle characteristics in women above age 43 with VER, ER and SR

	VER (13.5–15.5 mm)	ER (16.0–18.0 mm)	SR (18.5–20.5 mm)
Number of Patients	17	24	15
Average age (years)	44.9 ± 0.3	44.6 ± 0.3	45.0 ± 0.6
FSH (mIU/ml)	12.6 ± 3.3	9.3 ± 1.0	12.8 ± 5.9
AMH (ng/ml)	0.7 ± 0.2	0.8 ± 0.1	1.1 ± 0.3
P4/E2 ratio on hCG trigger day	2.3 ± 0.3	2.0 ± 0.3	2.1 ± 0.3
Retrieved oocytes	3.9 ± 0.6	5.6 ± 0.8	4.8 ± 1.0
Mature oocytes	2.0 ± 0.5	3.4 ± 0.5	2.4 ± 0.8
% of mature oocytes	53.5 ± 8.7	66.0 ± 5.5	51.1 ± 10.5
Immature oocytes	1.7 ± 0.4	1.4 ± 0.3	2.0 ± 0.8
% of immature oocytes	41.5 ± 8.2	22.8 ± 5.1	43.9 ± 10.5
Atretic oocyte/patient	0.3 ± 0.1	0.8 ± 0.3	0.3 ± 0.2
% of atretic oocyte	7.4 ± 3.6	11.2 ± 3.5	4.8 ± 2.6
Fertilized oocytes	2.3 ± 0.4	3.0 ± 0.5	2.9 ± 0.6
Total transferable embryos	2.0 ± 0.3	2.7 ± 0.4	2.3 ± 0.6
High quality embryos	0.9 ± 0.1^a^	2.3 ± 0.4^b^	0.7 ± 0.2^a^
% of high quality embryos	52.3 ± 8.7^a^	83.8 ± 5.8^b^	34.0 ± 13.2^a^
Pregnant rate	5.9% (1/17)^a^	16.7% (4/24)^b^	6.7% (1/15)^a^

VER, very early retrieval; ER, early retrieval; SR, standard retrieval; ^a/a^denotes no significant difference; ^a/b^denotes significant difference

Among the 37 patients with POA/oPOI, 24 were triggered with hCG at 14.0–18.0 mm (ER); the remaining 13, following SR, at 18.5- ≥20.5 mm. Patient assignment was again driven by patient location: Those undergoing monitoring at our center uniformly underwent ER, while long-distance patients, monitored distantly, usually had SR. Table 2 demonstrates that patient characteristics, including age, FSH and AMH, between the two groups did not differ.

**Table 2:** Patient population and IVF cycle characteristics of POA/oPOI patients

IVF Parameters	ER	SR
Number of patients	24	13
Age (years)	39.4 ± 0.6	38.5 ± 0.6
Serum FSH (mIU/ml)	12.8 ± 1.3	11.3 ± 1.2
Serum AMH (mg/ml)	0.49 ± 0.09	0.43 ± 0.12
P4/E2 ratio on trigger day	2.49 ± 0.37	3.16 ± 0.68
Retrieved oocytes	3.4 ± 0.6	5.7 ± 1.3
Matured oocytes	2.7 ± 0.44	3.4 ± 0.9
% of mature oocytes	81.5 ± 4.5	55.8 ± 8.3^a^
Immature oocytes	0.45 ± 0.1	0.7 ± 0.2
% of immature oocytes	12.6 ± 3.4	14.4 ± 5.1
Atretic oocyte	0.32 ± 0.2	1.3 ± 0.3^a^
% of atretic oocyte	7.9 ± 3.0	28.2 ± 7.2^a^
Fertilized oocytes	2.2 ± 0.4	3.1 ± 0.7
% of fertilized oocytes	87.5 ± 5.2	86.8 ± 5.1
Total transferable embryos	1.6 ± 0.2	2.5 ± 0.3
High quality embryos	1.1 ± 0.2	0.9 ± 0.2
% high quality embryos	68.2 ± 10.7	53.8 ± 9.3
Clinical pregnancy rate	41.7% (10/24)	7.7% (1/13)^a^

ER, early retrieva; SR, standard retrieval

Outcomes were similar, unless ^a^reflects significant difference (*P* < 0.05)

### Laboratory procedures

**Fig. 1:**
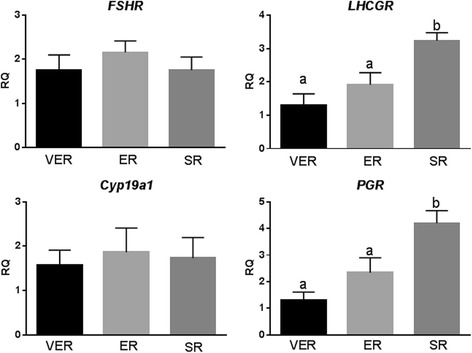
Gene expression at mRNA level in GCs with VER, ER and SR. *FSHR*, FSH receptor; *LHCGR*, LH receptor; *Cyp19a1*, P450 aromatase; *PGR*, progesterone receptor; ^a/a^denote no statistical difference; ^a/b^denote significant statistical difference (*P* < 0.05)

**Fig. 2:**
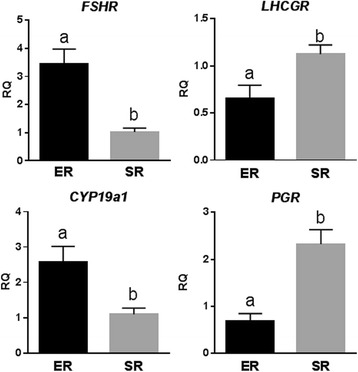
Gene expression at mRNA level in GCs of POA/oPOI patients with ER and SR. *FSHR*, FSH receptor; *LHCGR*, LH receptor; *Cyp19a1*, P450 aromatase; *PGR,* progesterone receptor; ER, early retrieval; SR, standard retrieval; ^a/b^denote significant statistical difference (*P* < 0.05)

All retrievals, embryo cultures and embryo transfers occurred at our center, where also granulosa cell (GC) isolation, GC cultues, RNA extraction and real-time PCR analyses were performed according to previously described protocols [3]. Specifically, gene expression at mRNA level in GCs were established for FSH receptor (*FSHR*), luteinizing hormone receptot (*LHCPR*), P450 aromatase (*CYP19a1*) and progesterone receptor (*PGR*). These genes are well-recognized markers of luteinization, as we described in great detail before. The same also applied RNA extraction and real-time PCR, which were in detail described in the predecessor study to this manuscript [3]. GCs were obtaimed at time of oocyte retrievals, and were until RNA isolation frozen at − 80 degrees (Figs. 1 and 2).

Each sample was run in duplicate. For each target gene the number of mRNA molecules was calculated and expressed relative to ribosomal protein L19 (RPL19) reference mRNA.

Oocytes with obvious first polar body were identified as mature (MII); those without as immature (GV and MI). Oocytes with brown dark color, cytoplasmic fragments and/or broken membranes were identified as atretic. Embryos with 4–12 blastomeres of equal size and with minimal cytoplasmic fragmentation were censured as “good” embryos, suitable for transfer [3].

Here reported pregnancies are clinical pregnancies, defined by visualization of at least a single sac on ultrasound containing a fetal heart at normal rate. Live birth outcomes were not determined in this study but are age dependent. In women above age 43, they can be expected to be approximately 50% of clinical pregnancy rates, with the other half of patients experiencing miscarriages (Gleicher N, et al., unpublished data).

### Statistics

Statistical comparisons were made with Prism software. Unpaired t-test with Welch’s correction was used for comparision of clinical data. One-way ANOVA, followed by the Tukey Test, was used to anylysize PCR results.

### Ethics approval by institutional review board (IRB) and data availability

Here reported studies were performed on anonymized tissues destined for disposal, and involved anonymized electronic research data sets of patients, who had given written consent to use of tissues and of their medical records for research purposes as long as their identity was protected and their medical record remained confidential. Here presented study was approved by the center’s IRB (CHR-IRB). Study data sets are deposited in the center’s IRB files, and can be requested by qualified investigators by contacting Ms. J. Tapper at (jtapper@thechr.com).

## Results

### Determination of best lead follicle size for hCG trigger

As Table 1 demonstrates, all three study groups were of similar and very advanced ages. Based on FSH and AMH levels, they also had similarly LFOR. All three groups also produced similar oocyte and embryo numbers. Also translating into improved clinical pregnancy rates, good quality embryos were significantly more prevalent in the ER group (*P* < 0.05), where pregnancy rates practically trippeled to 16.7% from 5.9% in the VER and 6.7% in the SR groups, respectively (*P* < 0.05; Table 1). The mean age of 44.6 ± 0.3 years of women in the ER group reemphasizes the remarkable clinical pregnancy rate in this patient group.

In order to confirm that timing of oocyte retrieval affects luteinization of follicles, we investigated luteinization-related gene expression in isolated GCs in vitro. As Fig. 1 demonstrates, in contrast to our earlier study in older women [3], *FSHR* and *Cyp19a1* were not significantly affected by follicle sizes at time of hCG trigger. *PGR* and *LHCGR*, two of the most important luteinization markers, did, however, again demonstrate significantly higher expression in SR than VET and ET (*P* < 0.05), confirming our prior report that, above age 43, earlier retrieval to significant degrees avoids PL. These data, however, also suggest that in POA/oPOI women PL may be less severe than in women above age 43.

Suggesting that PL with advancing age, indeed, occurs earlier and earlier, GCs of VER patients demonstrated in vitro the least evidence of premature PL. Embryo quality and clinical pregnancy rates in this VER group, however, were equaly poor as in the SR group, both being significantly lower than in the ER group (*P* < 0.05, Table 1). Though small patient numbers in each annual age group call for caution in not overinterpreting these results, other factors than PL may come into play with VER, which may negatively affect egg/embryo quality and clinical pregnancy chances. A possible suspect is insufficient cytoplasmatic maturation, a fairly typical findings in oocytes derived from small follicles [8], further discussed in the discussion section.

As of this point in our investigation, lead follicle sizes of 16.0–18.0 mm at time of hCG trigger, therefore, at all ages appear to results in best overall clinical pregnancy chances.

### Does lead follicle size matter in POA/oPOI?

Table 2 demonstrates that follicles of POA/oPOI patients biologically as well as clinically, indeed, behaved very similarly to previously reported older women above age 43 years [3]. More mature (81.5 ± 4.5 vs. 55.8 ± 8.3%, *P* < 0.05) and fewer atretic oocytes (7.9 ± 3.0% vs.28.2 ± 7.2%, *P* < 0.05) were obtained with ER than SR, while immature oocyte numbers were similar. These observation suggest PL of follicles with SR.

This was confirmed with in vitro GC studies, when luteinization-related gene expression in isolated GCs (Fig. 2), indeed, demonstrated similar findings, as recently reported in women above age 43 [3]: ER increased *FSHR* and *CYP19a1*, and decreased *LHCPR* and *PGR* expression. Like in older women, GCs from ER patients were, thus, significantly less luteinized than in women with SR.

Clinical pregnancies increased in ER patients in this study more than five-fold (41.7% vs. 7.7%, *P* < 0.05), even considerably exceeding previously observed improvements in women over age [3], and in this study observed improvement in older women above age 43 years.

Characteristics of the POA/oPOI patient population in which these results were obtained are important to reemphasize: Their mean age was 39.4 ± 0.6 years, their FSH was 12.8 ± 1.3mIU/mL and their AMH was 0.49 ± 0.09 ng/mL. They, thus, represented a POA/oPOI patient population with very significant degrees of LFOR. Here reported improvements in clinical pregnancy rate with ER, therefore, have to be viewed as quite remarkable. With SR, clinical pregnancy rates, in contrast, in younger women with POA/oPOI and in women above age 43 were, only 7.7% and 6.7%, respectively (Tables 1 and 2).

## Discussion

Here presented results expand on our center’s recent report that older (above age 43) women’s follicles to significant degrees demonstrate PL, if allowed to mature to traditional follicle sizes. Early oocyte retrieval, with hCG trigger at approximately 16 mm lead follicle sizes, in such patients, however, avoided oocyte exposure to PL, thereby significantly improving clinical pregnancy rates [3].

In this follow up study, we confirm that molecular evidence of PL declines with decreasing lead follicle size trigger (Fig. 1); yet, pregnancy rates did not follow in parallel. Indeed, too small and too big lead follicle sizes resulted in similarly poor clinical pregnancy rates, while a median follicle size, here described as ER (16.0–18.0 mm), significantly improved clinical pregnancy rates.

These findings are of interest because 16.0 mm was the follicle size arbitrarily chosen by us in our initial study of older women above age 43 years [3]. Here reported results, therefore, confirm that a lead follicle range of approximately 16.0–18.0 mm, overall, appears the best size to trigger ovulation with hCG in women with LFOR, whether due to advanced female age or POA/oPOI.

As Fig. 1 demonstrates, molecular evidence of PL, however, further declines with VER, yet IVF pregnancy rates remain poor. Another cause, therefore, must be responsible for here observed disappointing outcomes with VER. Though still to be confirmed experimentally, the most likely explanation is that, even if morphologically seemingly mature, too early retrieved oocytes, still, feature insufficiently matured cytoplasm [8].

Since completion of this study, we indeed, confirmed this hypothesis in preliminary investigations, when seeing geatly improved clinical pregnancy rates, and even one live birth, in women as old as almost 48 years with hCG triggers given at lead follicle sizes as small as 12 mm [9]. We attribute these outcome differences in comparison to here reported results with triggesr below 16 mm to attempts at enhancing cytoplasmic maturity by overnight culture of unstripped (from their cumulus cells) oocytes before fertilization (Gleicher et al., unpublished data).

Considering the mean age of 44.6 years (range 43–49) in here studied patient population, the clinical pregnancy rate in the ER group of 16.7% is especially noteworthy. Indeed, even the much lower rates of, respectively, 5.9% and 6.7% in VER and SR patients, at such advanced ages have to be considered excellent [2]. Assuming that such clinical pregnancy rates can be maintained in larger study populations, HIER through ER would offer an increasingly attractive clinical alternative to oocyte donation for at least a subgroup of older women who currently are only rarely still considered candidates for IVF with use of own eggs.

Our findings in POA/oPOI even exceed those in women above age 43: They, therefore, demonstrate that so affected younger women indeed “prematurely age” their ovaries, − as the molecular biology of their follicles mimics that of much older women. Like older women, POA/oPOI patients also apparently demonstrate speeded up oocyte maturation since ER does not increase the percentage of immature oocytes.

Molinari et al. recently demonstrated that “older” women but not cumulus cells from younger females with normal ovarian reserve activate gene pathways associated with hypoxia and oxidative stress [10]. Activation of these pathways in older women and younger women with POA/oPOI may, therefore, in similar ways lead to premature luteinization of follicles.

The improvement in clinical pregnancy rate with ER in women with POA/oPOI from 7.7% to 41.7% is, however, nothing but remarkable, and significantly exceeds outcome improvements obtained in older women [3]. In both studies, SR resulted in only a 7.7% pregnancy rate. In this study, however, ER improved clinical pregnancy outcomes in older women to 16.7% and in our initial study to a very similar 15.5% [3]. Yet, in POA/oPOI patients, the clinical pregnancy rate jumped over five-fold to to 41.7%. This observation demonstrates once more that youth always outperforms in IVF outcomes advanced female age. Moreover, PL in POA/oPOI may be less severe than at ages > 43 years.

When investigating a random population of younger women Kryou et al. did not find IVF outcome differences when triggering either under or above 16 mm lead follicle size [11]. Their patient population, however, likely did not include many, − if any, women with LFOR.

Because of limited numbers of study participants, here presented outcomes have to be considered as preliminary. They, however, nevertheless appear convincing in statistical power and congruency of results with our previously reported study in women above age 43 years [3]. Though prospectively randomized studies in follow up would be desirable, they have proven difficult to establish in women with LFOR, who are unlikely to agree to randomizations [12]. They are also supported by the fact that, only once we introduced HIER through age-specific ER into our center’s management schemed for older women, we established two pregnancies in the, likely, so-far oldest women to achieve this goal with use of their own eggs, − both only weeks removed from their 48th birthday [9].

It is surprising how little the literature has in recent years addressed lead follicle size at time of hCG trigger. Aside from already quoted studies, only two additional publications addressed this subject in the last 20 years, though none specifically in women with low functional ovarian reserve. Wittmaack et al... in a general IVF population concluded that for optimal outcomes, numbers of “adequate size” follicles at retrieval were more important than lead follicle size [13]. Mochtar et al, again in a general population, reported that in long agonist cycles IVF outcomes were superiror in women who were ovulation-induced at 22 mm lead follicle size rather than 18 mm [14].

It, of course, should not surprise that therapeutic interventions in older women with LFOR will be less effective than in younger women with LFOR. The same observation has, for example, also been made with androgen supplementation of LFOR [6]. Moreover, the ovarian environment in younger women is in generally less luteinized [15].

## Conclusion

We here expanded on our prior work, in which we demonstrated premature luteinization of follicles in women above age 43 [3] by demonstrating that similarly enhanced luteinization also occurs in younger women with POA/oPOI. Like older women, they, therefore, also benefit from early oocyte retrievals. In addition, we for the first time demonstrated that, in general, lead follicle sizes of 16-18 mm appear to represent the best range for hCG triggers. Because with advancing ages, as this study also demonstrated, PL further declines, HIER may vey well require even smaller trigger sizes in women at extremely older age (> 45 years). Still unpublished preliminary data indeed, appear to confirm this, as long as cytoplasmic maturity can be advanced in so early obtained oocytes.

## Abbreviations

AMH: Anti-Müllerian hormone
DHEA: Dehydroepiandrosterone
ER: Early oocyte retrieval
FSH: Follicle stimulating hormone
FSHR: FSH receptor
GCs: Granulosa cells
hCG: Human chorionic gonadotropin
HIER: Highly individualized egg retrieval
IRB: Institutional review board
IVF: In vitro fertilization
LFOR: Low functional ovarian reserve
LHCPR: Luteinizing hormone receptor
LOR: Low ovarian reserve
oPOI: Occult primary ovarian insufficiency
PGR: Progesterone receptor
PL: Premature luteinization
POA: Premature ovarian aging
SR: Standard retrieval
VER: Very early retrieval
